# COVID-19: Insight into the asymptomatic SARS-COV-2 infection and transmission

**DOI:** 10.7150/ijbs.48991

**Published:** 2020-08-27

**Authors:** Dongsheng Han, Rui Li, Yanxi Han, Rui Zhang, Jinming Li

**Affiliations:** 1 National Center for Clinical Laboratories, Beijing Hospital, National Center of Gerontology; Institute of Geriatric Medicine, Chinese Academy of Medical Sciences, P.R. China.; 2 Graduate School of Peking Union Medical College, Chinese Academy of Medical Sciences, Beijing, P.R. China.; 3 Beijing Engineering Research Center of Laboratory Medicine, Beijing Hospital, Beijing, P.R. China.

**Keywords:** SARS-COV-2, asymptomatic infection, serological assays, social distancing

## Abstract

The existence of a substantial but unclear number of asymptomatic SARS-COV-2 patients worldwide has raised concerns among global public health authorities. In this review, according to the published literature, we provided the evidence that asymptomatic infections can result in person-to-person transmission. Four studies suggested that the virus can be transmitted by asymptomatic patients for at least two consecutive generations, indicating its strong infectivity. Asymptomatic infection tends to be, but is not only, identified among young people (<20 years old). The majority of asymptomatic patients appear to have a milder clinical course during hospitalization, but the severity of the symptoms of the secondary patients infected by SARS-COV-2 from asymptomatic patients varies with their physical constitution. The proportion of asymptomatic individuals among all confirmed cases widely differed (from 1.95% to 87.9%) according to the study setting and the populations studied. The increasing large-scale tests are expected to give more information about the true number of asymptomatic infections in the population. In China and other countries, various guidelines for management of asymptomatic cases have been issued. Importantly, early detection, early reporting, early isolation and early treatment of asymptomatic patients require the joint efforts of policy makers, clinicians, technicians, epidemiologists, virologists and patients.

## Introduction

The COVID-19 pandemic caused by the SARS-CoV-2 virus is now straining or overwhelming health care systems worldwide. The clinical manifestations of COVID-19 are protean, including asymptomatic carrier, acute respiratory disease, and pneumonia of varying degrees of severity 1. Through the joint efforts of government administrations, academic institutions, medical workers, technology enterprises and ordinary community residents, the epidemic in China has been basically brought under control. However, in many countries, new infections and deaths are still increasing. As of July 4, 2020, over 11 million confirmed cases and more than 525,000 fatalities at a global scale have been attributed to COVID-19 infection. As the situation is rapidly evolving, it is unclear what the final scope and impact of this pandemic will be.

In addition to the potent transmissibility of the virus itself, there is increasing evidence that unnoticed, asymptomatic cases in the infected population may greatly accelerate the spread of SARS-COV-2 from person to person 2-4. Therefore, strict screening and management of patients with asymptomatic infection may be a key breakthrough to control the spread of the pandemic 5**.** Asymptomatic COVID-19 patients are those who carry the virus but do not show any symptoms (e.g., fever, gastrointestinal or respiratory symptoms), and no significant abnormalities on chest radiograph at time of laboratory confirmation 2, 6, 7. However, asymptomatic cases are difficult to identify because they seem to be normal except for the presence of viruses in their bodies, making the transmission caused by these silent patients difficult to prevent. Moreover, in the early stage of the outbreak, all medical resources tended to be used for the identification and management of critically ill patients, and asymptomatic patients have not attracted attention. These factors have led to a lack of systematic awareness of the prevalence and potential role of asymptomatic disease. Due to heightened concerns of the risks posed by these stealth virus carriers, China has published the numbers and conditions of asymptomatic people every day since April 1 and has further promulgated guidance for the management of asymptomatic COVID-19 cases nationwide 8. In this review, based on the findings of existing studies and reports, we summarize the clinical and epidemiological characteristics and discuss current screening and management strategies for asymptomatic patients. This knowledge is critical to design and implement efficient and globally coordinated interventions.

## What are the current findings regarding the clinical and epidemiological manifestations of asymptomatic infection?

According to recently published studies, we reviewed the clinical, virological and epidemiological characteristics of asymptomatic infections, which were usually determined by reverse transcription-polymerase chain reaction (RT-PCR) (**Table 1**). We found that (1) some asymptomatic cases at the time of the earliest test remained asymptomatic throughout the whole duration of laboratory and clinical monitoring, while others, which may account for approximately 80%, experienced clinical symptoms at a later stage of infection (presymptomatic patients) 9-12; (2) The viral load in respiratory specimens of asymptomatic patients was similar to that in symptomatic patients, ranging from 1×10^4^ to 1×10^7^ copies per milliliter 10, 13; (3) The period of positive nucleic acid tests of asymptomatic patients (the interval from the first day of positive nucleic acid tests to the first day of continuous negative tests) could be up to 3 weeks (ranging from 1-24 days) 14, 15; (4) Hoehl et al. successfully isolated SARS-CoV-2 from throat swabs of two asymptomatic patients in a cell culture of Caco-2 cells, suggesting the potential for presymptomatic transmission 16; (5) Increasing studies show clear epidemiological evidence of human-to-human asymptomatic spread of COVID-19 (described in the following section); (6) Asymptomatic infection tends to be, but is not only, identified among young people (<20 years old) 14, 15, 17-19; And (7) the majority (>90%) of asymptomatic patients appears to have a milder clinical course during hospitalization 15, but the severity of the symptoms of the secondary patients infected by SARS-COV-2 from asymptomatic patients varies based on their physical constitution 2, 20. These findings may be adjusted as the pandemic continues to unfold. With the accumulation of more research data, researchers will thoroughly clarify the occurrence, development and outcome of asymptomatic infections, providing reliable evidence for finalizing prevention, diagnosis and treatment strategies.

## Clear evidence of asymptomatic transmission

Asymptomatic transmission could be defined as the transmission of SARS-CoV-2 from an asymptomatic person (source or index patient) to a secondary patient, as ascertained by exposure and symptom onset dates, with no evidence that the secondary patient had been exposed to anyone else with COVID-19 4. Asymptomatic transmission is driven by two groups of patients, one with no self-perceived symptoms or clinically detectable signs throughout the 14 days of quarantine and the other in the incubation period (i.e., the time period between getting infected and showing symptoms) (presymptomatic transmission).

**Figure 1:**
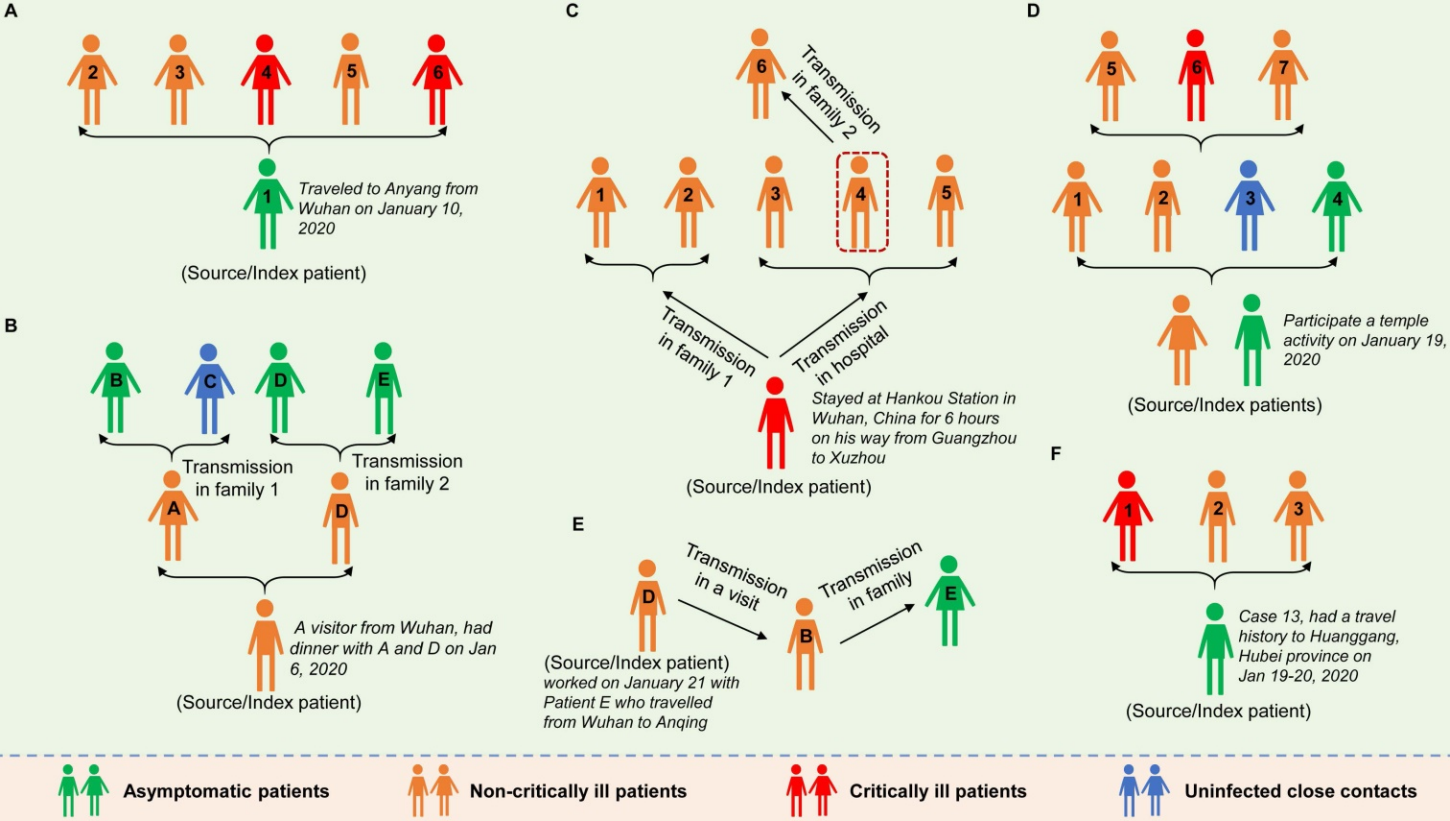
Six cluster outbreaks caused by asymptomatic transmission. The source/index patients transmitted SARS-COV-2 to close contacts during the asymptomatic period. The source/index patients may further develop symptoms (the severity of the disease is shown in different colors in the figure). The label (letter or number) on each person represents the person's ID in the original literature. The 6 cluster outbreaks are as follows: A is from reference 2, B is from reference 3, C is from reference 20, D is from reference 9, E is from reference 11, and F is from reference 14.

As early as January 2020, Chan et al. reported evidence of human-to-human transmission of SARS-CoV-2 in family settings, suggesting that asymptomatic patients might serve as a possible source to propagate the outbreak 17. By estimating the epidemiological data of distinct outbreak clusters from Singapore and Tianjin, China, Tindale et al. identified that the serial interval (i.e., the number of days between symptom onset in a primary case and a secondary case) was shorter than the incubation period of SARS-COV-2 by 2-4 days, supporting the likelihood that SARS-COV-2 viral shedding can occur in the absence of symptoms and before symptom onset 21. Similar findings were reported by other researchers 22-24. To date, epidemiologic studies have clearly documented SARS-CoV-2 transmission during the asymptomatic period. The evidence was mostly observed in cluster outbreaks, especially in family cluster outbreaks that occurred in China (**Figure 1**). In general, in these clusters, there is a clear history of contact between the source patients and the secondary patients; there is no other explanation for the infection; and according to the epidemiological survey, the source patients were asymptomatic when transmitting the virus to the secondary patients. For example, a study reported in January 2020 identified five patients with COVID-19 pneumonia infected by a 20-year-old asymptomatic woman (the source patient) who was thought to have acquired SARS-COV-2 infection from the epidemic center of Wuhan 2. This source patient showed no symptoms during the entire period of monitoring and isolation (**Figure 1A**). Tong et al. identified 2 COVID-19 patients (patients A and D) after their exposure to an asymptomatic person from Wuhan who was later confirmed to be positive for SARS-CoV-2 3. Patients A and D later transmitted SARS-CoV-2 to 3 family members (patients B, D and E) (**Figure 1B**). Li et al. confirmed asymptomatic and human-to-human transmission through close contacts in familial and hospital settings by analyzing the epidemiologic, laboratory, and clinical data of 7 COVID-19 patients in a 2-family cluster 20. The epidemiological survey showed that the source patient (56-year-old man) had stayed at Hankou Station in Wuhan, China for 6 hours on his way from Guangzhou to Xuzhou (**Figure 1C**). Qian and colleagues reported a family cluster of asymptomatic COVID-19 transmission in Zhejiang China 9. Two source patients became infected with SARS-COV-2 after a visit to a temple and then transmitted the virus to four other family members before experiencing symptoms. The four secondary patients then unknowingly (because they were asymptomatic) infected three other relatives through close contact (**Figure 1D**). Importantly, the latter three reports provided by Tong et al., Li et al. and Qian et al. together with another report from Luo et al. 11, showed that SARS-COV-2 can be spread through sustained transmission (at least two generations of spread) through asymptomatic/presymptomatic individuals (**Figure 1B-E**), suggesting that the virus has a strong infectivity. In another study in mainland China, the source patient (Case 13), who had a travel history to the city of Huanggang, Hubei province on Jan 19-20, 2020 and was diagnosed as an asymptomatic COVID-19 carrier, transmitted the virus to his cohabiting family members 14 (**Figure 1F**). In addition, the transmission of SARS-COV-2 infection from asymptomatic contacts has also been reported in other countries outside China, such as Germany 25, the USA 26 and Singapore 4.

In all the above reports, the source patients remained without any symptoms of infection during the clinical course (**Figure 1A, D and F**), or developed varying degrees of pneumonia (**Figure 1B, C and E**). Through comprehensive analysis of 24 asymptomatic COVID-19 patients screened among close contacts, the authors reported that asymptomatic carriers among the close contacts were prone to mild illness during admission. However, the communicable period could last as long as 21 days, and the communicated patients could develop severe illness 14. In four clusters of COVID-19 in Singapore, asymptomatic transmission occurred approximately 1-3 days before the source patient developed symptoms 4.

Since asymptomatic transmission is an established fact, how many individuals in the infected population are attributed to asymptomatic transmission? Several studies have made preliminary assessments. For example, using available data of 468 confirmed COVID-19 cases compiled from online reports from 18 provincial Centers for Disease Control and Prevention (CDC) in China, Du et al. identified that presymptomatic transmission might be responsible for 12.6% (59) of confirmed cases 23. In the Singapore outbreak, 6.4% (10/157) of locally acquired cases were attributed to presymptomatic transmission 4. Another study published by scientists in Belgium and the Netherlands showed that up to 48% (95% CI: 32-67%) of the 91 included COVID-19 cases in Singapore and 62% (95% CI: 50-76%) of the 135 included COVID-19 cases in Tianjin, China contracted the infection from someone who was presymptomatic 27.

## Studies on the proportion of asymptomatic infections

Now that the transmission of SARS-COV-2 through asymptomatic patients via person-to-person contact has been confirmed, reliable estimations of the proportion of asymptomatic infections among all COVID-19 patients are crucially needed to guide public health policy, especially to clarify the intensity and range of social distancing strategies to be implemented to prevent the spread of COVID-19 12. Robert Redfield, director of the US CDC, warned that up to a quarter of COVID-19 patients may not display any symptoms 28. We summarized the data of currently published literature. Overall, the detected asymptomatic proportion widely differed (from 1.95% to 87.9%) in the available studies with more than 10 confirmed individuals (Table 1). This situation is similar to the reported asymptomatic proportion of MERS-CoV (another coronavirus that infects humans) infections, which is varied from 12.5% to 91.7% based on the populations studied 29. Although reliable comparison may not be possible due to the differences in the study setting and the included populations, it can be seen, in general, that the asymptomatic proportions of SARS-COV-2 infections reported in China (<16%) and Singapore (<5%) are lower than those reported in Japan (>30%), Iceland (>40%) and the United States (>50%) (**Table 1**).

According to the National Health Commission (NHC) update, as of April 14, a total of 6,764 asymptomatic infections were reported nationwide in China, close to 8% of all confirmed SARS-COV-2 cases (approximately 83,000 cases) 31. In pediatric patients (<16 years of age) with COVID-19, a nationwide epidemiologic survey in China showed that 12.9% (94/731) of cases were asymptomatic 7. In Wuhan city, 15.8% (27/117) of diagnosed children did not exhibit any symptoms of infection or radiologic features of pneumonia during the course of hospitalization 33. The latest implemented universal SARS-CoV-2 testing in all pregnant patients presenting for delivery in New York City revealed that at this point in the pandemic, 87.9% of the patients who were positive for SARS-CoV-2 at delivery were asymptomatic, underscoring the risk of COVID-19 among asymptomatic obstetrical patients 34. Several studies suggested that the vast majority of cases (approximately 80%) that were asymptomatic at the time of testing would eventually develop into symptomatic cases 10-12, highlighting the importance of timely detection, treatment and management of asymptomatic patients.

However, most of the above results were not obtained by random sampling or testing all individuals in the population, but mainly through the analysis of screening data from high-risk groups (i.e., individuals who have symptoms, have traveled to areas infected with the virus or have been in contact with infected patients). Thus, these early results might not accurately reflect the real situation of the entire population. The large-scale population tests launched in increasing countries/regions are expected to reveal the true number of SARS-COV-2 infections in the population and the contribution of asymptomatic individuals. Recently, Iceland reported the results of its population screening based on nucleic acid testing, and found that 43% of the participants who tested positive had no symptoms 76. Importantly, before large-scale testing begins, full consideration should be given to the rationality of the research design, the standardization of the experimental operation, and the reliability of the quality of the reagents/kits. This is the premise to ensure the reliability of the final results.

## The role of nucleic acid and serological assays in the diagnosis of asymptomatic infection

The current first-line laboratory diagnostic tests for COVID-19 worldwide include nucleic acid testing (mainly RT-PCR) and rapid serological antibody (mainly IgM and IgG) testing. As the transmission of SARS-COV-2 may occur in the early course of infection and a high viral load in respiratory samples could be detected 13, RT-PCR testing for this virus is more suitable for screening at earlier stages of infection in key populations, such as patients with obvious symptoms and close contacts of asymptomatic patients 35. However, due to various factors (such as nonstandard sampling, inadequately trained personnel, unqualified reagents, etc.), false negative results in nucleic acid testing are almost inevitable 36. It has been reported that the detection rate of currently available RT-PCR methods is between 30% and 60% 37-38. The issues contributing to false negative results in nucleic acid assays include insufficient viral load in infected persons, nonstandard sampling and handling, imperfect sensitivity of in vitro diagnostic reagents, and inadequate clinical laboratory testing capabilities 39. For some cases, multiple samples and repeated testing may be required to obtain a final diagnosis 40. In addition, in the early stages of outbreaks, many countries did not have sufficient reagents to carry out large-scale testing, which led to nucleic acid testing being more inclined to be performed on persons with obvious symptoms and reduced the detection for asymptomatic infections 41. In theory, nucleic acid testing will miss individuals who have been asymptomatic for past infections (convalescent cases). These reasons make the true number of infected individuals, including asymptomatic individuals, in the population is currently unclear.

Detecting serum antibodies is an effective supplement to nucleic acid assays. Data show that the positive rate can be increased from 51.9%, for a single RT-PCR test, to 98.6% when combining an IgM antibody assay with an RT-PCR test 42. Serological assays are rapidly being developed and have proven to be useful in confirming COVID-19 infection 35, 43. In view of its simple operation and low cost, serological detection is suitable for large-scale screening 41. A study showed that the median time of IgM antibody response was 5 days (interquartile range [IQR], 3-6), while the appearance of IgG antibody occurred on day 14 (IQR, 10-18) after symptom onset 42. Thus, serological testing is unlikely to play an important diagnostic role in the early stage of SARS-COV-2 infection, but is more suitable in retrospective investigations for accurately determining the burden of infection, the contribution of asymptomatic infection, the basic reproduction number and total mortality, which have important implications for public health 44, 45. Eran et al. carried out serologic testing for SARS-CoV-2 antibodies in 3,330 people in Santa Clara County, Northern California, suggesting that the actual number of infected people may be 50- to 85-fold the reported number of confirmed cases 41. However, the reliability of antibody kits they used was been questioned by some scientists because of the lack of rigorously assessment. On April 10-14, a random seroprevalence survey of 865 residents in Los Angeles County, California suggested that only 4.06% (35/865) of the tested people have SARS-COV-2 antibodies, suggesting that most people are still susceptible to contracting COVID-19 46. At present, large-scale serological screening projects are running in many countries and regions 47-49, if the study design is rigorous (full consideration is given to variation factors such as sampling, research population, reagent quality, etc.), the results of which will have important guiding significance for understanding the true number of asymptomatic infections and controlling the current disease outbreak worldwide.

## Management of asymptomatic patients: call for public cooperation

Asymptomatic cases are easily neglected in disease screening and epidemic prevention. The asymptomatic COVID-19 patients who have been identified include close contacts of COVID-19 cases during their medical observation period, individuals involved in cluster outbreaks, people who were unknowingly exposed to infected patients and were identified when the infection source was being traced, and individuals who recently traveled to areas/countries/regions with high infection rates (i.e., imported cases) 14, 18. However, in reality, there may be more undetected asymptomatic patients. These cases may not be aware that they have been infected and therefore will not actively self-isolate or seek medical attention 50, or they may be misdiagnosed during the screening and thus transmit the virus to others unknowingly. This situation complicates fighting the pandemic, forcing healthcare workers around the world to shift their focus from containing the infection to mitigation 51. As the large-scale domestic epidemic has passed, one of China's current core tasks is to strictly screen, report and manage patients with asymptomatic infections to prevent possible future outbreaks. The actions China takes next will provide experience for the rest of the world to respond to the subsequent SARS-COV-2 outbreak. On April 08, 2020, China's State Council issued guidance for the management of asymptomatic COVID-19 cases 8. Briefly, the guidelines call for standardized reporting of asymptomatic cases, which means that all healthcare facilities should report asymptomatic cases detected by them within 2 hours through direct online reporting. County-level CDCs are required to complete a case investigation within 24 hours after receiving the report of an asymptomatic case and report all the close contacts of the case. Then, the asymptomatic case and all the close contacts need to undergo a 14-day centralized quarantine for medical observation. Similarly, 14 days of active monitoring or quarantine for contacts of asymptomatic cases are also recommended by other countries 12, 52, 53**.** Once the individuals complete the 14-day quarantine and test negative twice in a row with one test every 24 hours, they are released from medical observation. Those who show no symptoms and still test positive will be kept under centralized medical observation. If asymptomatic cases display clinical manifestations during the period of centralized medical observation, they shall be immediately transferred to designated medical institutions for standardized treatment in accordance with the confirmed cases 54. Furthermore, the guidance also pointed out that those who are released from centralized quarantine still need to be under medical observation for another fortnight. This strategy is useful in monitoring the risk of possible recurrence of the virus in recovered COVID-19 patients 55, 56. These series of management measures for asymptomatic patients in China are very strict compared to those of other countries. In the United States and the United Kingdom, asymptomatic patients might discontinue isolation if they still have no symptoms within one week of the first positive COVID-19 diagnostic test 57, 58.

There are currently no proven pharmaceutical treatments (vaccines or antivirals). Rigorous implementation of traditional public health measures such as isolation and quarantine, social distancing and community containment is now the preferred option to reduce transmission of the virus 59, 60. Since the SARS-COV-2 outbreak began, China has implemented all these nonpharmaceutical measures, such as case isolation, contact tracing and quarantine, physical distancing and community containment, with unprecedented efforts and successfully curbed the spread of the virus across the country in a relatively short period of time 24, 61. Other countries are also aware of the necessity of implementing such measures. Once community transmission is detected, implementing the combined intervention of isolating infected individuals and their family members (including voluntary isolation at home); closing schools, factories or office buildings; suspending public markets; cancelling gatherings; and even locking-down affected cities are recommended 51, 53, 59, 62-64. Related studies have also suggested the importance of these measures in reducing SARS-CoV-2 spread, including the spread from asymptomatic individuals 32, 65-67.

Furthermore, for personal protection, wearing masks is another important public health intervention for limiting the spread of respiratory pathogens. As transmission from asymptomatic infected individuals has been documented for COVID-19, wearing masks in public places would help to intercept the transmission and prevent the spread by these apparently healthy infectious sources (asymptomatic patients) 68-70. Other suggestions should be accepted as well, such as restricting movement in affected cities, avoiding close contact with patients who are symptomatic, avoiding unnecessary gatherings, and washing hands frequently (with soap and water for at least 20 s) 70-72. When coming into contact with confirmed/suspicious patients, people should proactively inform health authorities and seek testing to determine whether they are infected.

## Conclusions

Addressing COVID-19 is now an urgent health and social concern. However, controlling the pandemic only by detecting, isolating and treating symptomatic or critically ill patients is not sufficient because accumulating evidence has confirmed the asymptomatic transmission of SARS-COV-2. Several studies have even suggested that the virus can be transmitted by asymptomatic patients for at least two consecutive generations, indicating its strong infectivity. Therefore, it is of great public health significance to screen and manage asymptomatic patients and their close contacts via multiple strategies to contain potential outbreaks. Several scattered studies have evaluated the contribution of asymptomatic patients in the population. However, there is no comparability between their results due to the differences in the distribution and size of the studied populations, so the true scale of asymptomatic infections remains unclear. Ongoing large-scale population screening is expected to answer this question. Studies have provided a cautionary warning that SARS-COV-2 virus may be transmitted through multiple routes in addition to the most common respiratory droplet spread. It is necessary to fully consider the sample type and sampling time to improve the detection rate. In addition, a combination of molecular and serological tests is needed to identify virus carriers when necessary. Asymptomatic patients themselves should complete the standard isolation and treatment process as required by governments and doctors. Additionally, when considering that the spread of SARS-COV-2 may occur through respiratory droplets, contact with contaminated objects or fecal viral shedding, which is currently being investigated 4, 73, individuals should also pay attention to personal hygiene and reduce close contact with healthy people to avoid causing new infections. In short, early detection, early reporting, early isolation and early treatment of asymptomatic patients require the joint efforts of policy makers, clinicians, technicians, epidemiologists, virologists and patients.

## Figures and Tables

**Table 1:** Studies evaluating the asymptomatic proportion among SARS-COV-2-infected patients

ID	Case description	Total number of studied cases	Total number of patients with SARS-COV-2 infection	Number (%) of asymptomatic patients at the time of testing	Number of asymptomatic patients who developed symptoms	Number of asymptomatic patients who never showed any symptoms	Reference
1	All cases in mainland China as of February 11, 2020	72,314	45,561^#^	889 (1.95%)	-	-	30
2	Nationwide pediatric patients with COVID-19 in China from January 16 to February 8, 2020	2,143	731	94 (12.9%)	-	-	7
3	Cases on board the Diamond Princess cruise ship, Yokohama, Japan, 2020	3,063	634	328 (51.7%)	-	113.3 (95%CrI: 98.2-128.3)*	12
4	All cases of COVID-19 reported in Singapore from January 23 to March 16,2020	243	243	10 (4.12%)	-	-	4
5	Close contacts of COVID-19 patients in Ningbo City, China as of March 16, 2020	2,147	191	30 (15.7%)	-	-	74
6	SARS-CoV-2 Infection in children in Wuhan City, China from January 28 to February 26, 2020	1,391	171	27 (15.8%)	-	-	33
7	Diagnosed patients in Chongqing City, China from January 2020 to March 2020	167	167	20 (12.0%)	-	-	18
8	Residents of Vo' city, Italy	5,155	102	42 (42.5%)	-	-	75
9	Residents of Iceland	13,080	100	43 (43.0%)	-	-	76
10	Diagnosed patients in Anqing City, China as of February 21, 2020	83	83	8 (9.6%)	7	1	11
11	Residents of a Large Homeless Shelter in Boston	829	41	27(65.9%)	-	-	77
12	All pregnant patients presenting for delivery in New York City from March 22 to April 4, 2020	215	33	29 (87.9%)	3	26	34
13	Residents in a longterm care skilled nursing facility in King County, Washington	76	23	13 (56.5%)	10	3	10
14	Japanese citizens evacuated from Wuhan City, China	565	13	4 (30.8%)	-	-	50
15	German travelers returning from Wuhan City, China	126	2	2 (100%)	-	-	16

^#^ Included 44,672 symptomatic patients and 889 asymptomatic patients; * Estimated by a statistical model.

## Abbreviations

CDC: Centers for Disease Control and Prevention
IQR: Interquartile range
RT-PCR: reverse transcription-polymerase chain reaction

## References

[1] Lai CC, Liu YH, Wang CY, Wang YH, Hsueh SC, Yen MY (2020). Asymptomatic carrier state, acute respiratory disease, and pneumonia due to severe acute respiratory syndrome coronavirus 2 (SARS-CoV-2): Facts and myths. J Microbiol Immunol Infect 2020. in press. [https://doi.org/10.1016/j.jmii.

[2] Bai Y, Yao L, Wei T, Tian F, Jin D, Chen L (2020). Presumed Asymptomatic Carrier Transmission of COVID-19. JAMA.

[3] Tong Z, Tang A, Li K, Li P, Wang H, Yi J (2020). Potential Presymptomatic Transmission of SARS-CoV-2, Zhejiang Province, China, 2020. Emerg Infect Dis.

[4] Wei WE, Li Z, Chiew CJ, Yong SE, Toh MP, Lee VJ (2020). Presymptomatic Transmission of SARS-CoV-2- Singapore, January 23-March 16, 2020. MMWR. Morbidity and Mortality Weekly Report.

[5] Gandhi M, Yokoe DS, Havlir DV (2020). Asymptomatic Transmission, the Achilles' Heel of Current Strategies to Control Covid-19. N Engl J Med.

[6] Surveillances V (2020). The Epidemiological Characteristics of an Outbreak of 2019 Novel Coronavirus Diseases (COVID-19)—China, 2020. China CDC Weekly.

[7] Dong Y, Mo X, Hu Y, Qi X, Jiang F, Jiang Z Epidemiology of COVID-19 Among Children in China. Pediatrics 2020: e20200702.

[8] TheStar Media Group (2020). China imposes new rules on detection of asymptomatic cases. 2020. https://www.thestar.com.my/news/regional/2020/04/12/china-imposes-new-rules-on-detection-of-asymptomatic-cases. Date last accessed: 14 April.

[9] Qian G, Yang N, Ma AHY, Wang L, Li G, Chen X (2020). A COVID-19 Transmission within a family cluster by presymptomatic infectors in China. Clin Infect Dis.

[10] Kimball A, Hatfield KM, Arons M, James A, Taylor J, Spicer K (2020). Asymptomatic and Presymptomatic SARS-CoV-2 Infections in Residents of a Long-Term Care Skilled Nursing Facility-King County, Washington, March 2020. MMWR. Morbidity and Mortality Weekly Report.

[11] Luo SH, Liu W, Liu ZJ, Zheng XY, Hong CX, Liu ZR (2020). A confirmed asymptomatic carrier of 2019 novel coronavirus (SARS-CoV-2). Chin Med J (Engl).

[12] Mizumoto K, Kagaya K, Zarebski A, Chowell G (2020). Estimating the asymptomatic proportion of coronavirus disease 2019 (COVID-19) cases on board the Diamond Princess cruise ship, Yokohama, Japan, 2020. Euro Surveill.

[13] Zou L, Ruan F, Huang M, Liang L, Huang H, Hong Z (2020). SARS-CoV-2 Viral Load in Upper Respiratory Specimens of Infected Patients. New Engl J Med.

[14] Hu Z, Song C, Xu C, Jin G, Chen Y, Xu X (2020). Clinical characteristics of 24 asymptomatic infections with COVID-19 screened among close contacts in Nanjing, China. Sci China Life Sci.

[15] Wang Y, Liu Y, Liu L, Wang X, Luo N, Li L (2020). Clinical Outcomes in 55 Patients With Severe Acute Respiratory Syndrome Coronavirus 2 Who Were Asymptomatic at Hospital Admission in Shenzhen, China. J Infect Dis.

[16] Hoehl S, Berger A, Kortenbusch M, Cinatl J, Bojkova D, Rabenau H (2020). Evidence of SARS-CoV-2 Infection in Returning Travelers from Wuhan, China. N Engl J Med.

[17] Chan JF, Yuan S, Kok KH, To KK, Chu H, Yang J (2020). A familial cluster of pneumonia associated with the 2019 novel coronavirus indicating person-to-person transmission: a study of a family cluster. Lancet.

[18] Tao Y, Cheng P, Chen W, Wan P, Chen Y, Yuan G (2020). High incidence of asymptomatic SARS-CoV-2 infection, Chongqing, China. medRxiv.

[19] Song H, Xiao J, Qiu J, Yin J, Yang H, Shi R (2020). A considerable proportion of individuals with asymptomatic SARS-CoV-2 infection in Tibetan population. medRxiv.

[20] C L, F J, L W, L W, J H, M D (2020). Asymptomatic and Human-to-Human Transmission of SARS-CoV-2 in a 2-Family Cluster, Xuzhou, China. Emerg Infect Dis.

[21] Tindale L, Coombe M, Stockdale JE, Garlock E, Lau WYV, Saraswat M (2020). Transmission interval estimates suggest pre-symptomatic spread of COVID-19. medRxiv.

[22] Nishiura H, Linton NM, Akhmetzhanov AR (2020). Serial interval of novel coronavirus (2019-nCoV) infections. medRxiv.

[23] Du Z, Xu X, Wu Y, Wang L, Cowling BJ, Meyers LA (2020). Serial Interval of COVID-19 among Publicly Reported Confirmed Cases. Emerg Infect Dis.

[24] Zhao S, Gao D, Zhuang Z, Chong M, Cai Y, Ran J (2020). Estimating the serial interval of the novel coronavirus disease (COVID-19): A statistical analysis using the public data in Hong Kong from January 16 to February 15, 2020. medRxiv.

[25] Rothe C, Schunk M, Sothmann P, Bretzel G, Froeschl G, Wallrauch C (2020). Transmission of 2019-nCoV Infection from an Asymptomatic Contact in Germany. New Engl J Med.

[26] Holshue ML, DeBolt C, Lindquist S, Lofy KH, Wiesman J, Bruce H (2020). First Case of 2019 Novel Coronavirus in the United States. New Engl J Med.

[27] Ganyani T, Kremer C, Chen D, Torneri A, Faes C, Wallinga J (2020). Estimating the generation interval for COVID-19 based on symptom onset data. medRxiv.

[28] Rich Haridy (2020). CDC Director warns 25 percent of COVID-19 cases may present no symptoms. 2020. https://newatlas.com/health-wellbeing/covid-19-cases-contagious-asymptomatic-presymptomatic-cdc-director/. Date last accessed: 01 April.

[29] Wang Y, Tong J, Qin Y, Xie T, Li J, Li J (2020). Characterization of an asymptomatic cohort of SARS-cov-2 infected individuals outside of wuhan, china. Clin Infect Dis.

[30] Wu Z, McGoogan JM (2020). Characteristics of and Important Lessons From the Coronavirus Disease 2019 (COVID-19) Outbreak in China: Summary of a Report of 72 314 Cases From the Chinese Center for Disease Control and Prevention. JAMA 2020. in press. [https://doi.org/ 10.1001/jama.

[31] XINHUANET (2020). Asymptomatic COVID-19 cases reach 6,764 on Chinese mainland. 2020. http://www.nhc.gov.cn/xcs/fkdt/202004/f2b50e681e7042f8bef2abf2029ffa13.shtml. Date last accessed: 15 April.

[32] Day M Covid-19: identifying and isolating asymptomatic people helped eliminate virus in Italian village. BMJ 2020: m1165.

[33] Lu X, Zhang L, Du H, Zhang J, Li YY, Qu J (2020). SARS-CoV-2 Infection in Children. N Engl J Med.

[34] Sutton D, Fuchs K, D Alton M, Goffman D (2020). Universal Screening for SARS-CoV-2 in Women Admitted for Delivery. New Engl J Med.

[35] Zhang W, Du RH, Li B, Zheng XS, Yang XL, Hu B (2020). Molecular and serological investigation of 2019-nCoV infected patients: implication of multiple shedding routes. Emerg Microbes Infect.

[36] Wikramaratna P, Paton RS, Ghafari M, Lourenco J (2020). Estimating false-negative detection rate of SARS-CoV-2 by RT-PCR. medRxiv.

[37] Al-Tawfiq JA, Memish ZA (2020). Diagnosis of SARS-CoV-2 Infection based on CT scan vs. RT-PCR: Reflecting on Experience from MERS-CoV. J Hosp Infect.

[38] Ai T, Yang Z, Hou H, Zhan C, Chen C, Lv W Correlation of chest CT and RT-PCR testing in coronavirus disease 2019 (COVID-19) in China: A report of 1014 cases. Radiology 2020: 200642.

[39] Tahamtan A, Ardebili A (2020). Real-time RT-PCR in COVID-19 detection: issues affecting the results. ExpertRev Mol Diagn.

[40] Jung YJ, Park G, Moon JH, Ku K, Beak S, Kim S (2020). Comparative analysis of primer-probe sets for the laboratory confirmation of SARS-CoV-2. bioRxiv.

[41] Bendavid E, Mulaney B, Sood N, Shah S, Ling E, Bromley-Dulfano R (2020). COVID-19 Antibody Seroprevalence in Santa Clara County, California. medRxiv.

[42] Guo L, Ren L, Yang S, Xiao M, Chang D, Yang F (2020). Profiling Early Humoral Response to Diagnose Novel Coronavirus Disease (COVID-19). Clin Infect Dis.

[43] Li Z, Yi Y, Luo X, Xiong N, Liu Y, Li S (2020). Development and clinical application of a rapid IgM-IgG combined antibody test for SARS-CoV-2 infection diagnosis. J Med Virol.

[44] Petherick A (2020). Developing antibody tests for SARS-CoV-2. The Lancet.

[45] Vogel G (2020). New blood tests for antibodies could show true scale of coronavirus pandemic. Science news. 2020. https://www.sciencemag.org/news/2020/03/new-blood-tests-antibodies-could-show-true-scale-coronavirus-pandemic. Date last accessed: 19 March.

[46] Sood N, Simon P, Ebner P, Eichner D, Reynolds J, Bendavid E (2020). Seroprevalence of SARS-CoV-2-Specific Antibodies Among Adults in Los Angeles County, California, on April 10-11, 2020. JAMA.

[47] Jon Cohen. Unprecedented nationwide blood studies seek to track U.S. coronavirus spread. 2020. https://www.sciencemag.org/news/2020/04/unprecedented-nationwide-blood-studies-seek-track-us-coronavirus-spread. Date last accessed: 7 April, 2020

[48] Gretchen Vogel. 'These are answers we need.' WHO plans global study to discover true extent of coronavirus infections. Science news. 2020. https://www.sciencemag.org/news/2020/04/these-are-answers-we-need-who-plans-global-study-discover-true-extent-coronavirus. Date last accessed: 2 April, 2020

[49] KYODO NEWS (2020). China launches widespread survey to tackle asymptomatic virus cases. 2020. https://english.kyodonews.net/news/2020/04/5f56b79eb592-china-launches-widespread-survey-to-tackle-asymptomatic-virus-cases.html. Date last accessed: 15 April.

[50] Nishiura H, Kobayashi T, Suzuki A, Jung S, Hayashi K, Kinoshita R (2020). Estimation of the asymptomatic ratio of novel coronavirus infections (COVID-19). Int J Infect Dis.

[51] Parodi SM, Liu VX (2020). From Containment to Mitigation of COVID-19 in the US. JAMA 2020. in press. [https://doi.org/10.1001/jama.

[52] CDC (2020). Interim US Guidance for Risk Assessment and Public Health Management of Persons with Potential Coronavirus Disease 2019 (COVID-19) Exposure in Travel-associated or Community Settings. 2020. https://www.cdc.gov/coronavirus/2019-ncov/hcp/guidance-risk-assesment-hcp.html. Date last accessed: 2 March.

[53] Ng Y, Li Z, Chua YX, Chaw WL, Zhao Z, Er B (2020). Evaluation of the Effectiveness of Surveillance and Containment Measures for the First 100 Patients with COVID-19 in Singapore - January 2-February 29, 2020. MMWR. Morbidity and mortality weekly report.

[54] National Health Commission & State Administration of Traditional Chinese Medicine Diagnosis, Treatment Protocol for Novel Coronavirus Pneumonia (Trial Version 7) (2020). 2020. https://www.chinadaily.com.cn/pdf/2020/1.Clinical.Protocols.for.the.Diagnosis.and.Treatment.of.COVID-19.V7.pdf. Date last accessed: 3 March.

[55] Lan L, Xu D, Ye G, Xia C, Wang S, Li Y (2020). Positive RT-PCR Test Results in Patients Recovered from COVID-19. JAMA.

[56] Li Y Hu Y Zhang X Yu Y Li B Wu J [Follow-up testing of viral nucleic acid in discharged patients with moderate type of 2019 coronavirus disease (COVID-19)] Zhejiang Da Xue Xue Bao Yi Xue Ban 2020 49 10 10.3785/j.issn.1008-9292.2020.03.11 PMC8800664 32391676

[57] CDC (2020). Discontinuation of Isolation for Persons with COVID-19 Not in Healthcare Settings (Interim Guidance). 2020. https://www.cdc.gov/coronavirus/2019-ncov/hcp/disposition-in-home-patients.html. Date last accessed: 3 May.

[58] GOV.UK (2020). Stay at home: guidance for households with possible coronavirus (COVID-19) infection. 2020. https://www.gov.uk/government/publications/covid-19-stay-at-home-guidance/stay-at-home-guidance-for-households-with-possible-coronavirus-covid-19-infection#ending-isolation. Date last accessed: 28 April.

[59] Wilder-Smith A, Freedman DO (2020). Isolation, quarantine, social distancing and community containment: pivotal role for old-style public health measures in the novel coronavirus (2019-nCoV) outbreak. J Travel Med.

[60] Ferretti L, Wymant C, Kendall M, Zhao L, Nurtay A, Abeler-Dörner L Quantifying SARS-CoV-2 transmission suggests epidemic control with digital contact tracing. Science 2020: b6936.

[61] Wang C, Liu L, Hao X, Guo H, Wang Q, Huang J (2020). Evolving Epidemiology and Impact of Non-pharmaceutical Interventions on the Outbreak of Coronavirus Disease 2019 in Wuhan, China. medRxiv.

[62] ECDC (2020). Coronavirus disease 2019 (COVID-19) pandemic: increased transmission in the EU/EEA and the UK-seventh update. 2020. https://www.ecdc.europa.eu/en/publications-data/rapid-risk-assessment-coronavirus-disease-2019-covid-19-pandemic. Date last accessed: 25 March.

[63] Davies NG, Kucharski AJ, Eggo RM, Gimma A, Edmunds WJ (2020). The effect of non-pharmaceutical interventions on COVID-19 cases, deaths and demand for hospital services in the UK: a modelling study. medRxiv.

[64] Ray D, Salvatore M, Bhattacharyya R, Wang L, Mohammed S, Purkayastha S (2020). Predictions, role of interventions and effects of a historic national lockdown in India's response to the COVID-19 pandemic: data science call to arms. medRxiv.

[65] Chao DL, Oron AP, Srikrishna D, Famulare M (2020). Modeling layered non-pharmaceutical interventions against SARS-CoV-2 in the United States with Corvid. medRxiv.

[66] Koo JR, Cook AR, Park M, Sun Y, Sun H, Lim JT (2020). Interventions to mitigate early spread of SARS-CoV-2 in Singapore: a modelling study. Lancet Infect Dis.

[67] Hellewell J, Abbott S, Gimma A, Bosse NI, Jarvis CI, Russell TW (2020). Feasibility of controlling COVID-19 outbreaks by isolation of cases and contacts. Lancet Glob Health.

[68] Leung CC, Lam TH, Cheng KK (2020). Mass masking in the COVID-19 epidemic: people need guidance. Lancet.

[69] Cohen J (2020). Not wearing masks to protect against coronavirus is a 'big mistake,' top Chinese scientist says. Science 2020. https://www.sciencemag.org/news/2020/03/not-wearing-masks-protect-against-coronavirus-big-mistake-top-chinese-scientist-says. Date last accessed: 27 March.

[70] Ma Q, Shan H, Zhang H, Li G, Yang R, Chen J (2020). Potential utilities of mask-wearing and instant hand hygiene for fighting SARS-CoV-2. J Med Virol.

[71] Jin YH, Cai L, Cheng ZS, Cheng H, Deng T, Fan YP (2020). A rapid advice guideline for the diagnosis and treatment of 2019 novel coronavirus (2019-nCoV) infected pneumonia (standard version). Mil Med Res.

[72] CDC (2020). Preventing the Spread of COVID-19 in a Variety of Settings Throughout Your Community. Atlanta, GA: US Department of Health and Human Services, 2020. https://www.cdc.gov/coronavirus/2019-ncov/php/open-america/infection-control.html. Date last accessed: 20 April.

[73] Xu Y, Li X, Zhu B, Liang H, Fang C, Gong Y (2020). Characteristics of pediatric SARS-CoV-2 infection and potential evidence for persistent fecal viral shedding. Nat Med.

[74] Chen Y Wang AH Yi B Ding KQ Wang HB Wang JM The epidemiological characteristics of infection in close contacts of COVID-19 in Ningbo city Chinese Journal of Epidemiology 2020 41 000 10.3760/cma.j.cn112338-20200304-00251 32447904

[75] Lavezzo E, Franchin E, Ciavarella C, Cuomo-Dannenburg G, Barzon L, Del VC (2020). Suppression of a SARS-CoV-2 outbreak in the Italian municipality of Vo'. Nature.

[76] Gudbjartsson DF, Helgason A, Jonsson H, Magnusson OT, Melsted P, Norddahl GL (2020). Spread of SARS-CoV-2 in the Icelandic Population. New Engl J Med.

[77] Baggett TP, Keyes H, Sporn N, Gaeta JM (2020). Prevalence of SARS-CoV-2 Infection in Residents of a Large Homeless Shelter in Boston. JAMA.

